# Nicotine dependence, awareness of smoking-related health risks and readiness to quit among smoker patients in Government Medical College, Kannur, India: A cross-sectional study

**DOI:** 10.18332/tpc/207354

**Published:** 2025-07-25

**Authors:** Swetha Manoj

**Affiliations:** 1 Government Medical College, Kannur, India

**Keywords:** smoking cessation, smoking, nicotine dependence, nicotine addiction, smoking related health issues

**Dear Editor,**

India is one of the largest tobacco producers and the second-highest consumer of tobacco globally^1^. Tobacco use remains a major preventable risk factor for cancer, chronic respiratory disease, cardiovascular disease, and stroke. An estimated 1.35 million deaths occur annually in India due to tobacco use^2^.

We conducted a cross-sectional study among 200 male smoker inpatients at Government Medical College, Kannur, Kerala, between September 2021 and September 2022. Our objective was to assess nicotine dependence, awareness of smoking-related health problems, and readiness to quit smoking.

A semi-structured questionnaire was used to conduct bedside interviews. The Fagerström test for nicotine dependence (FTND) assessed dependence levels as low (0–3), medium (4–6), and high (7–10). Readiness to quit was categorized using the Transtheoretical Model (TTM), which categorizes individuals into the quitting stages: pre-contemplation, contemplation, preparation, action, and maintenance. Awareness was assessed across 15 tobacco-related health issues, with participants scoring 0 (no awareness) to 2 (high awareness) for each of them. Data were coded, entered into Excel, and analyzed using descriptive statistics and inferential methods, including Fisher’s exact test. A p-value <0.05 was considered statistically significant.

Medium nicotine dependence (44%) was most prevalent, followed by low (38%) and high (18%) levels. Awareness was highest for lung cancer (76.5%), oral cancer (65%), and heart disease (62%), yet only moderate for stroke (51.5%), chronic bronchitis (52.5%), passive smoking consequences (lung cancer 53.5%, chronic bronchitis 52.5%). Awareness was markedly lower for reproductive health effects – infertility (27.5%), low birth weight (27%), and female genital cancer (29%) – as well as bladder cancer (35.5%).

Regarding readiness to quit, 47% were in the action stage, 18% in preparation, and 10.5% in maintenance. Our analysis showed that participants with higher knowledge levels had significantly lower nicotine dependence (p<0.05). Moreover, those with higher awareness were more likely to be in preparation, action, or maintenance stages of quitting.

Though respiratory and cardiovascular conditions had relatively higher awareness, many were still recognized by only half of the participants – indicating room for improved education. Awareness of less-publicized effects like reproductive outcomes remained inadequate.

Our findings are consistent with national-level data: A meta-analysis (2010–2022) reported an overall tobacco usage prevalence of 35.2% in Indian adults^3^. In a 2019–2021 national study, 38% of men consumed tobacco and 27% tried to quit^4^. Quit attempts were notably lower among individuals with limited education and from economically disadvantaged groups^5,6^. Chellappa et al.^7^ similarly noted higher nicotine dependence and less intention to quit among less educated and lower socioeconomic populations.

Our findings underscore the need for targeted awareness campaigns, particularly on under-recognized risks like reproductive health effects. Expanding cessation services and stricter tobacco control policies, could improve quit rates. Future longitudinal studies are essential to measure the long-term impact of such interventions.

**Table 1:** Nicotine dependence, awareness of smoking-related health problems, stage of readiness to quit smoking, a cross-sectional study among inpatient smokers in Government Medical College, Kannur, India, September 2021 – September 2022 (N=200)

*Characteristics*	*n*	*%*
**Nicotine dependence level**		
Low (0–3)	76	38
Medium (4–6)	88	44
High (7–10)	36	18
**Health problem**		
Lung cancer	153	76.5
Oral cancer	130	65
Heart diseases	124	62
Atherosclerosis	107	53.5
Stroke	103	51.5
Chronic bronchitis	105	52.5
Bladder cancer	71	35.5
Female genital cancer	58	29
Infertility	55	27.5
Low birth weight in neonates	54	27
Passive smoking related lung cancer	107	53.5
Passive smoking related chronic bronchitis	105	52.5
Mean lifetime is shorter for a smoker	96	48
Diseases reversible after smoking cessation	90	45
Lifetime augmentation after smoking cessation	88	44
**Stage of readiness to quit smoking**		
Pre-contemplation	13	6.5
Contemplation	36	18
Preparation	36	18
Action	94	47
Maintenance	21	10.5

## Supplementary Material



## Data Availability

The data supporting this research are available from the author on reasonable request.
