# Supplementary material for: Nicotine dependence, awareness of smoking-related health risks and readiness to quit among smoker patients in Government Medical College, Kannur, India: A cross-sectional study
Source: Tob Prev Cessat. 2025 Jul 25;11:10.18332/tpc/207354. doi: 10.18332/tpc/207354 (PMC12290922; doi:10.18332/tpc/207354)
Supplement: Supplementary file 1 [file TPC-11-35-s1.pdf]

SUPPLEMENTARY TABLE 1; Level of knowledge and severity of nicotine dependence among 200 inpatient smokers in Government Medical College, Kannur, Kerala. A Cross-sectional Study conducted between September 2021-September 2022

			**SEVERITY OF NICOTINE DEPENDENCE**		
			**low**	**medium**	**high**
**LEVEL OF KNOWLEDGE**	**0-20 (not satisfactory)**	**Count**	**31**	**57**	**14**
		**% within knowledge**	**30.4%**	**55.9%**	**13.7%**
	**21-30 (satisfactory)**	**Count**	**45**	**31**	**22**
		**% within knowledge**	**45.9%**	**31.6%**	**22.4%**

There is significant association between level of knowledge and severity of nicotine dependence. Smokers with satisfactory level of knowledge had low severity of nicotine dependence

SUPPLEMENTARY TABLE 2; Level of knowledge and current stage of readiness to quit smoking among 200 inpatient smokers in Government Medical College, Kannur, Kerala. A Cross-sectional Study conducted between September 2021-September 2022

			**LEVEL OF KNOWLEDGE**	
			**0-20 (not satisfactory)**	**21-30 (satisfactory)**
**CURRENT STAGE OF READINESS TO QUIT**	**Pre-contemplation**	**Count**	**9**	**4**
		**% within**	**69.2%**	**30.8%**
	**Contemplation**	**Count**	**23**	**13**
		**% within**	**63.9%**	**36.1%**
	**Preparation**	**Count**	**16**	**20**
		**% within**	**44.4%**	**55.6%**
	**Action**	**Count**	**45**	**49**
		**% within**	**47.9%**	**52.1%**
	**Maintenance**	**Count**	**9**	**12**
		**% within**	**42.9%**	**57.1%**

There is significant association between level of knowledge and current stage of readiness to quit. Smokers with satisfactory knowledge were in preparatory, action and maintenance stage. Smokers with not satisfactory knowledge were in precontemplation and contemplation stage.

© 2025 Manoj S.
